# Cultivated meat manufacturing: Technology, trends, and challenges

**DOI:** 10.1002/elsc.202300227

**Published:** 2023-11-20

**Authors:** Marline Kirsch, Jordi Morales‐Dalmau, Antonina Lavrentieva

**Affiliations:** ^1^ Cultimate Foods UG Berlin/Göttingen Germany; ^2^ Institute of Technical Chemistry Leibniz University of Hannover Hannover Germany

**Keywords:** cell selection, cultivated meat, mammalian cell expansion, media composition, regulatory challenges

## Abstract

The growing world population, public awareness of animal welfare, environmental impacts and changes in meat consumption leads to the search for novel approaches to food production. Novel foods include products with a new or specifically modified molecular structure, foods made from microorganisms, fungi, algae or insects, as well as from animal cell or tissue cultures. The latter approach is known by various names: “clean meat”, “in vitro meat” and “cell‐cultured” or “(cell‐)cultivated meat”. Here, cells isolated from agronomically important species are expanded ex vivo to produce cell biomass used in unstructured meat or to grow and differentiate cells on scaffolds to produce structured meat analogues. Despite the fast‐growing field and high financial interest from investors and governments, cultivated meat production still faces challenges ranging from cell source choice, affordable expansion, use of cruelty‐free and food‐grade media, regulatory issues and consumer acceptance. This overview discusses the above challenges and possible solutions and strategies in the production of cultivated meat. The review integrates multifaceted historical, social, and technological insights of the field, and provides both an engaging comprehensive introduction for general interested and a robust perspective for experts.

**Abbreviations**
AMP: Adenosine monophosphate
FDA: Food and Drug Administration
UN: United Nations
AMR: antimicrobial resistant
ECM: extracellular matrix
TERT: Telomerase Reverse Transkriptase
c‐MYC: cellular Myelocytomatosis
KRAS: Kirsten Rat Sarcoma
GMO: genetically modified organism
CHO: Chinese hamster ovary
ESCs: embryonic stem cells
iPSCs: induced pluripotent stem cells
MSCs: mesenchymal stem cells
PPARγ: Peroxisome proliferator‐activated receptor gamma
SMCs: satellite muscle cells
FCS: fetal calf serum
STR: stirred tank reactor
CFD: Computational Fluid Dynamics
PGA: poly(glycolic acid)
PEG: polyethylenglycol
IBMX: 3‐isobutyl‐1‐methylxanthine

## 1 A HISTORY OF MEAT‐EATING AND ORIGIN OF CULTIVATED MEAT TECHNOLOGY

The history of meat‐eating by humans began over 2,600,000 years ago with the use of sharp‐edge tools to cut through animal carcasses. Between 8000 BC and 5400 BC, humans domesticated animals such as pigs and chickens as constant, easily accessible sources of meat. The consumption of meat developed culinary traditions and preferences among the different cultures and civilizations, and the development of trade routes led to the exchange of various meats, spices, and cooking techniques, further diversifying meat consumption. With the industrialization and advancements in agriculture and transportation in more recent centuries, humans increased meat production and accessibility.

**FIGURE 1:**
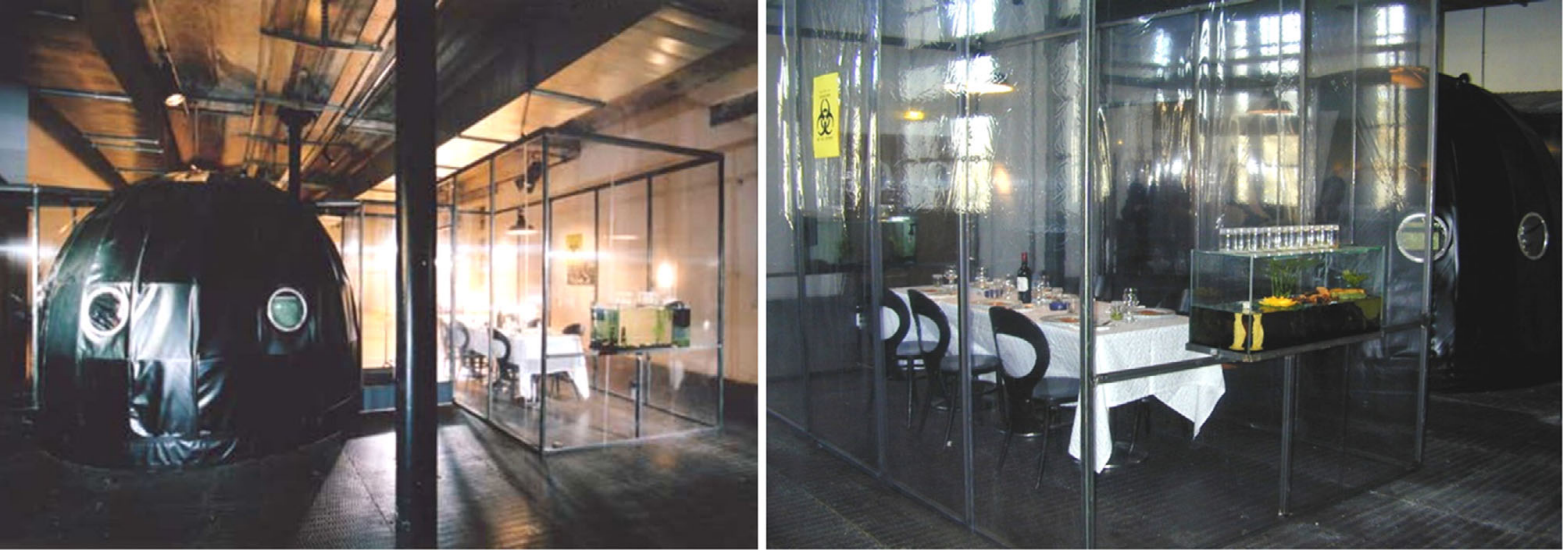
Disembodied kitchen art project installation with cell cultivation room connected to the dining room with a terrarium with frogs from which muscle cells were isolated. The Tissue Culture and Art project, https://tcaproject.net/portfolio/disembodied‐cuisine/

In 1894 French chemist Pierre Eugen Marcellin Berthelot claimed that by 2000 meat, milk and eggs could be synthesized in factories, and in 1930 Frederick Smith, the British Secretary for India, envisioned the emergence of “self‐replicating steaks”, where “from a ‘parent’ steam of exquisite tenderness, it will be possible to grow a steak as large and juicy as one could desire”[1]. In 1982 the first veggie burger was created and marked in the UK by Gregory Simms, in 1995 FDA approved the use of in vitro techniques for commercial meat production and in 1999 the first patent was filed for in vitro meat production in industrial scale [2, 3, 4]. Not only the food industry became interested in meat grown in the lab (now called “cultivated meat”): In 2003, artists and researchers Oron Catts and Ionat Zurr presented their art project “Disembodied Cuisine” as part of the L'Art Biotech exhibition in France, in which they explored the possibility of growing victimless meat using cell culture [5, 6]. In this project, biopsied skeletal muscles of frogs were grown on biopolymer scaffolds, while the healthy frogs lived next to them as part of the installation. On the final day of the exhibition, the steak was cooked and eaten for dinner and the four rescued frogs were released into a pond in the local garden (Figure 1). This art project marks the historical event of the first in vitro cultivated meat ever eaten by humans.

In 2004, the non‐profit organization New Harvest was created to increase awareness of cultivated meat early on by advertising, supporting networking and raising philanthropic donations to fund early‐stage research. In 2009, Professor Mark Post of Maastricht University stated in an interview with The New Yorker in 2011 that it would already be possible to breed burgers in the laboratory today if the appropriate financial support were available. This was followed by initially anonymous funding from Google co‐founder Sergey Brin, which allowed the world's first in vitro cultivated burger to be cooked and served at a globally broadcast media event. The production cost of this burger was 300,000 euros and although animal cell culture supplements (fetal calf serum, FCS) were used to grow the cells, it was demonstrated that the production of cultured meat is scientifically possible [3, 7, 8]. In 2016, several organizations and startups appeared, such as the Good Food Institute, Mosa Meats (founded by Mark Post), Memphis Meats (today known as UPSIDE Foods) and Modern Meadow. In 2016, Memphis Meats showcased a cultivated meatball at a tenth of the price of Mark Posts burger patty, and in 2019, Future Meat announced cultivated chicken at <500€/lb [9].

Today, a growing number of companies (more than 100 worldwide) are working to commercialize and scale cultivated meat production. Even traditional agriculture companies started to invest in cultivated meat (e.g., 100 M€ buyout from JBS to BioTech company in Spain or Cargill's investment in Aleph Farm or Memphis meat) [10, 11]. Most of these companies are focused on cultivated beef, followed by poultry, pork, seafood and exotic meats (e.g., mammoth, kangaroo and horse), most of them are located in North America, followed by Asia and Europe [12].

In light of the blossoming popularity of cultivated meat, we have borne witness to many historical moments during the last years. In 2020, East Just, after successfully passing regulatory approval in Singapore, sold in the restaurant “1880” the first cell‐cultivated chicken nugget and made Singapore the first country to ever allow the purchase and consumption of in vitro cultivated meat [13, 14, 15, 16]. During the writing of this article, in June 2023, subsequent to the Food and Drug Administration's (FDA) authoritative pronouncement in November 2022 affirming the safety of cultivated meat for human consumption, the United States Department of Agriculture (USDA) granted its endorsement for the retail sale of “cultivated” chicken to UPSIDE Foods and Good Meat (a division of Eat Just), making the US only the second country in the world to allow commercial sales of in vitro cultivated meat products [17]. This regulatory approval represents a momentous stride towards the widespread commercialization of lab‐grown meat, thereby opening up access to a consumer base exceeding 400 million individuals [18, 19].

Cellular agriculture for cultivated meat production has become a fast‐growing branch of the biotechnology and food industry, addressing important issues related to animal welfare, population growth, environmental and health issues, as well as sustainability aspects of conventional industrial livestock farming.

## 2 MAJOR MOTIVATION IN CULTIVATED MEAT MANUFACTURING

**FIGURE 2:**
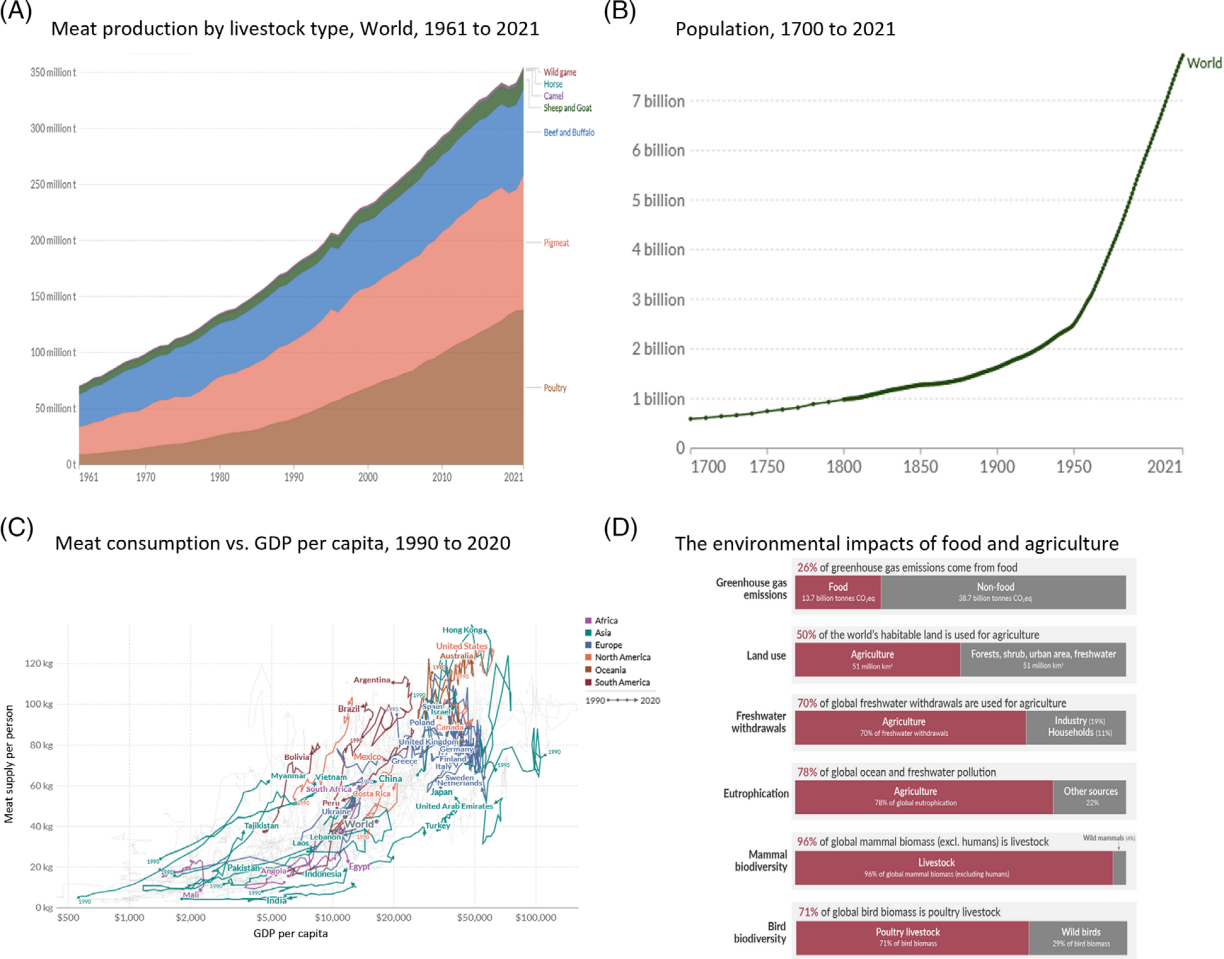
(A) Meat production worldwide in recent years. (B) Population growth from 1700 to 2021 demonstrates rapid increase over the last centuries. (C) Meat consumption per capita shows a correlation with gross domestic product. (D) Environmental impacts of food and agriculture. https://ourworldindata.org

Since the first domesticated animals, livestock farming has grown into a huge industry with a total production of over 350 million tons of meat per year worldwide (Figure 2A). The current system, which allows the production of a high quantity of affordable meat due to intensive factory farming and very high subsidies, is being challenged by the climate crisis, public awareness of animal welfare as well as the pandemic experience of zoonoses and new consumer needs [12, 20–24].

### 2.1 Environmental sustainability

According to the United Nations, the global human population reached 8 billion people at the end of 2022 and is expected to reach 9 billion by 2050 (Figure 2B) [25]. As the global population grows and global trends show that richer countries consume more meat per capita (Figure 2C), the Food and Agriculture Organization of the United Nations (FAO) predicts that animal meat consumption will increase by more than 70% by 2050 compared to 2010 [26, 27]. A further increase in demand for meat‐based diets would consequently require a further expansion of the current livestock system. However, compared to plant‐based products, animal‐based products, especially the beef industry, already have a larger environmental footprint regarding greenhouse gas (GHG) and nitrogen emissions, land and water usage and biodiversity loss (Figure 2D) [28, 29, 30, 31, 32].

For instance, livestock production accounts for almost 20% of all anthropogenic greenhouse gas emissions, twice as large as the whole plant‐based sector and makes animal production by far the largest emission contributor in the food system [33, 34, 35]. Additionally, livestock accounts for over 20% of the total terrestrial animal biomass and three quarters of the Earth's cultivated area (and 30% of total land surface) is used alone for livestock and their needs [36]. Since up to 97% of the calories animals consume in food are lost to essential metabolic pathways as well as the production of non‐edible tissues, the question arises whether the large agricultural areas used for livestock food production would not be more effectively used to feed the global population directly [37, 38, 39, 40]. In 2020, Heinke et al. estimated that 41% of total agricultural water use (blue and green water) is used annually solely for the production of livestock feed [41]. Furthermore, since livestock farming is the main cause of deforestation and one of the main causes of soil degradation, pollution, climate change, overfishing, sedimentation in coastal areas and the introduction of invasive species, it is one of the most important contributors to biodiversity reduction [42]. Considering these factors, it is very questionable whether a further expansion of livestock farming in the future would even be realistic or particularly sustainable. Even potential improvements for future conventional livestock production are likely to be offset by the increased demand, making the development of alternative technologies moving away from traditional agriculture crucial [28].

### 2.2 Animal welfare

Global meat consumption and production has already more than quadrupled in the last 60 years (Figure 2A). Such a strong increase in demand for meat has created pressure on the meat industry to optimize their production processes which consequently also leads to compromises in animal husbandry. Factors such as market value, land and sustained resources are crucial for the livestock industry. In order to remain economically competitive, the welfare and health of the animals raised is neglected and animal welfare measures are reduced to a minimum [43, 44, 45]. For instance, Ben‐Arye et al. stated that raising a chicken comparable to a pet would cost up to three times more than the product cost made from the animal itself [46]. Especially in the case of livestock for slaughter, health is considered to be of secondary importance and animals often develop diseases caused by, for example, nutrient deficiencies or stress [47, 48]. The competition between animal welfare and economy in the intensive factory farming industry can also result in it being cheaper to let animals suffering from diseases die or slaughter them instead of providing treatment [49, 50, 51].

In recent decades, however, the public view has changed and farm animals are now increasingly seen and respected as sentient beings with physical and psychological needs and moral and ethical questions regarding intensive factory farming are raised. Accordingly, over the last decades, many laws and guidelines have been adapted or enacted in favor of animal welfare [52]. However, despite strict guidelines, these are often not sufficiently enforced at national level and still lead to numerous cases of animal cruelty (traumatic mutilations, amputations, and castrations without anesthesia) [53]. Altogether, for these reasons, global interest in alternatives to animal‐based products has grown significantly and research into alternative agricultural technologies is increasing worldwide [54].

Especially cellular agriculture seems to have the potential to provide a long‐term cruelty‐free and authentic alternative to conventional animal‐based products [55]. However, animal welfare must also be ensured here. Although the United Nations (UN) announced in 2022 that cell‐based food production methodologies are characterized and ready to move to production facilities, the world's first approved in vitro cultivated meat product on the market was not animal‐slaughter‐free [56, 57]. In addition to a cruelty‐free methodology to obtain cells, an animal component‐free media should be used.

### 2.3 Meat consumption behavior

Due to climate and animal welfare concerns, Generation Y (Millennials) and Generation Z are increasingly concerned about food sustainability [58]. Moreover, as they become parents, they pass on their eating habits and their beliefs to their children [59]. These are current and future generations seeking for alternatives to animal‐based products who provide a theoretical consumer base for cellular agriculture products. In general, more and more people, especially in the younger generations, identify themselves as flexitarians (1/3 of the US population) and more than half of the world's households use plant‐based food already as a dietary foundation. Next to sustainability and animal welfare as driving factors for consumers to change to plant‐based diets, especially health concerns play a key role for consumers to reduce their meat consumption. One concern are the dietary properties of meat—it is claimed that meat is not good for health because meat is high in saturated fats and cholesterol, which leads to coronary heart disease and higher mortality [60]. People who care about their health usually consume less red meat but would be interested in meat that retains its flavor but has a healthier fat composition. Despite growing consumer health and safety concerns, sub‐optimal taste and texture of alternative meat products are among the biggest factors influencing consumer meat consumption. Many meat eaters are reducing their meat consumption, but cannot imagine switching completely to alternative products. This would require an alternative product with the same taste experience and texture as conventional meat.

### 2.4 Public health

Additional to the elimination of nutrition‐related diseases, is the elimination of foodborne diseases a current and crucial topic. In particular, the COVID‐19 pandemic has made the importance of infection awareness apparent, since 75% of new infectious diseases in humans are zoonotic (originating from animal sources). Further increase in livestock farming will lead to additional destruction of wildlife habitats, which has a direct effect on the increase of zoonotic diseases. Furthermore, intensive livestock farming together with limited animal welfare measures lead to foodborne diseases (e.g., ebola or swine and avian influenza), the spread of pathogenic microorganisms (e.g., salmonella, E.coli or campylobacter) and a rise of antibiotic resistance (70%–80% of antibiotics sold worldwide are used in livestock) [8, 9]. Extensive usage of antibiotics in livestock farming can result in the selection of antimicrobial resistant (AMR) strains. Over the next 20 to 30 years, AMRs are estimated to be responsible for more deaths than cancer and to cost member countries of the Organization for Economic Co‐Operation and Development (OECD) several trillion dollars annually [61].

### 2.5 One of the solutions: Cultivated meat

Today, attempts are being made to rethink the production of agricultural products, especially meat and meat substitutes. One of the solutions is “cultivated meat”: a sustainable alternative for consumers who want to be more responsible but don't want to change the composition of their diet. Cultivated meat aims to replace conventional meat production with the help of cell and tissue culture [14]. Cultivated meat is expected to have numerous benefits compared to conventional animal agriculture: (1) it allows the reduction of needed resources in terms of nutrition, land and water use, (2) the reduction of greenhouse gas emission, (3) elimination of zoonotic infections, and (4) antibiotic resistances. Important to note that various naming is used for this approach, among others are (1) in vitro meat, (2) (cell‐)cultivated or (cell‐)cultured meat, (3) clean meat, and (4) cell‐based meat [58].

### 2.6 Cultivated meat concepts

#### 2.6.1 Meat components

Meat consists primarily of muscle cells, followed by connective tissue cells (e.g., fibroblasts), fat cells (adipocytes), blood vessels and extracellular matrix (ECM) (Table 1). Muscle cells form bundles called fibers. Fat cells grow in small or large deposits of adipose tissue (depending on species, breed, sex and age), which affect meat marbling [62]. Cells produce an ECM composed of proteins, proteoglycans, and glycoproteins that supports tissue and cell growth and enables homeostasis. In muscle tissue, the ECM is also responsible for tissue elasticity and cell alignment [46, 63]. As an energy‐hungry tissue, muscle is highly vascularized, making blood a very important compound of meat in terms of color and flavor. Meat consists of 75% of water, 19% of protein, 2.5% of fat, 1.2% of carbohydrates and 1.65% of nitrogen compounds [64]. In addition to the nutritional and safety aspects, the quality and acceptability of the meat strongly depend on its organoleptic properties: appearance, aroma, taste and texture. Major flavor molecules in meat are amino acids, hemoproteins, sulphur and carbonyl compounds, short peptides and lipids [46].

**TABLE 1:** Major meat components and their function in vivo and contribution to the meat properties.

Meat component	Initial function	Culinary contribution to the final meat	Cultivated meat component
Muscle	Contraction/movement	Texture, biomass	Muscle cells
Fat	Storage of energy participates in signaling	Contribution of flavor substances, juiciness, texture	Adipocytes
Connective Tissue	Structure, Support, protection	Texture, biomass	Non/Fibroblasts
ECM	Structure, tissue homeostasis/cell‐matrix interactions	Texture, juiciness	Natural and synthetic Scaffolds
Blood vessels	Oxygen and nutrients supply, metabolite removal	Color, flavor	Recombinant heme proteins/food dye/beet juice

#### 2.6.2 Possible approaches

To date, several approaches have been developed to create alternative meat. The **protein‐based approach**, which includes plant and insect proteins, is used to produce meat surrogates. Here, an extrusion process is used in which proteins are simultaneously mixed, kneaded, and rapidly heated under pressure to produce extrudates with a meat‐like texture. The production of protein‐based meat involves three steps: (1) protein extraction and modification, (2) formulation by adding food‐grade adhesives, vegetable fat and flour, and (3) processing—a protein reshaping process to create a meat‐like texture [1]. To improve the organoleptic properties of protein‐based meat, innovative technologies are being developed applying recombinant protein additives, shear cell technology and 3D printing [1].

The **cell‐based approach** involves cell biomass expansion with final scaffold‐free formulation to alternative meat [1]. Here, stem cells or differentiated cells are isolated from animal tissues and grown in cell culture flasks or in bioreactors to achieve a desired biomass. The **fiber‐based** approach was developed by the Post working group in Maastricht—here hundreds of muscle cells self‐organize into fibers, forming a muscle/meat‐like structure [7]. The **tissue‐based** approach represents the most complex technique for in vitro meat production. In this technique, different cell types are cultured on edible scaffolds to produce meat‐like constructs.

#### 2.6.3 Tissue engineering approaches in cultivated meat production

**FIGURE 3:**
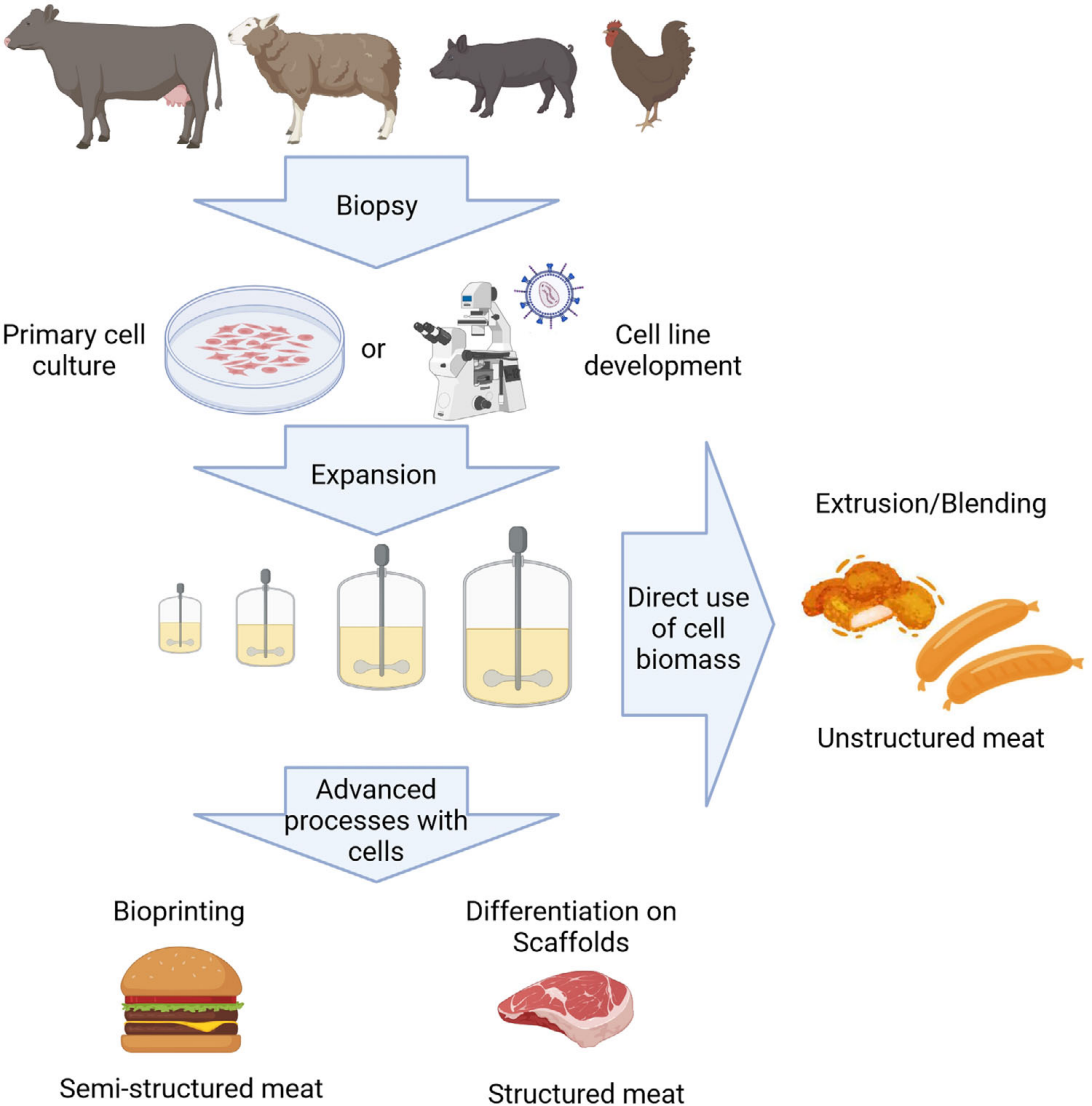
Cultivated meat production pipeline. Created with Biorender.

The idea and concept of growing cells outside of the animal organism is very old—cell culture technique has over 100 years history [65]. To create **structured cultivated meat**, many researchers use the state‐of‐the‐art principles of tissue engineering, originally developed for regenerative medicine. However, in medicine, engineered tissues are being developed and optimized under very strict good manufacturing practices (GMP) and in terms of biological functionality, biodegradability, and cell survival after implantation. These goals are not aimed at the production of cruelty‐free inexpensive cultivated meat. Rather, organoleptic and nutritional properties in a very affordable and sustainable manner are now in the foreground. The tissue engineering approach involves four major steps for tissue manufacturing: (1) biopsy, (2) cell isolation, (3) cell expansion and (4) cell differentiation on scaffolds (Figure 3). The same approach can be used for cultivated meat manufacturing: here biopsy or postmortem tissue sample can be taken to isolate cells, which are expanded in flasks or bioreactors, seeded on edible scaffolds, and differentiated. Similar to the different requirements for the quality of the end product compared to regenerative medicine, different strategies and challenges must also be considered during the production process, which are discussed below.

## 3 CULTIVATED MEAT PRODUCTION CHALLENGES

**FIGURE 4:**
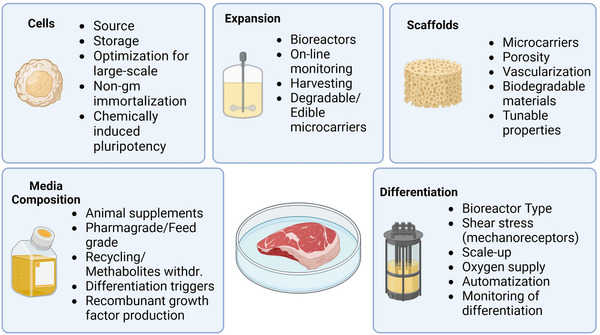
Optimization areas and relevant parameters in cultivated meat production. Created with Biorender.

There are five main areas of optimization in cultivated meat production: (1) cell selection and optimization, (2) media composition, (3) expansion strategies, (4) differentiation protocols, and (5) scaffold materials used (Figure 4). For cultivated meat production cells are grown in a two‐step process—expansion and differentiation. In each process step, some experience from cell culture and tissue engineering can be adopted, but new challenges also arise.

### 3.1 Cell selection and optimization

The selection of cells for cultivated meat production is influenced by several factors, including the target animal species, the desired end‐product and regional regulations. The choice of cells ranges from different types of stem cells to fully differentiated adult somatic cells. Relevantly, since the goal is producing large‐scale biomass at affordable cost, it is crucial to select fast‐growing cells. Muscle and fat cells are essential components of meat and both cell types can be isolated from adult tissues and grown ex vivo. However, the population doubling times of these cells are relatively high and the Hayflick limit is reached after a few divisions when the cells enter an irreversible growth arrest state, defined as replicative senescence [66].

There are several challenges when selecting cell lines. Firstly, companies often claim to isolate cells from live animals through non‐painful biopsies. However, to have a good starting cell population, tissue samples larger than typical biopsy samples are required. In order to obtain larger cell numbers, there are three main strategies: direct isolation of big cell numbers, cell immortalization and selection of fast‐growing cells. The first strategy, aiming the isolation of high amounts of differentiated cells from the tissues of sacrificed animals immediately after death, however, does not solve existing challenges since: (1) it requires larger numbers of slaughtered animals and (2) these cells are highly limited in their ex vivo proliferation capacity. The second strategy is the creation of immortalized, endlessly renewable cell banks. However, primary isolated adult stem cells are not immortal; they do have a limited number of in vitro population doublings. To immortalize these cells there are some strategies, for instance by **introducing specific oncogenes or telomerase reverse transcriptase gene** (TERT, c‐MYC, KRAS, HOXA9, SV40) into their genome. Such manipulation, however, turns cells into GMOs, which is not acceptable in most cases, as mentioned above. Another way to immortalize cells is the treatment with **chemical carcinogens** or **ionizing radiation**. Newly, researchers from Hebrew University published their study on **spontaneous immortalization** of chicken fibroblasts after long‐term in vitro cultivation [67]. The rate of such spontaneously immortalized cells is very low – 2% of all cell population. Spontaneous immortalization was earlier often detected in cells isolated from small animals/rodents (e.g., CHO or SmcMF, NIH‐3T3 cells). In big animals, as well as in humans, spontaneous immortalization is an extremely rare event [68]. So far, bovine mammary epithelial cells (BME65Cs) are the only established and studied spontaneously immortalized cell line [69]. The rest of the livestock cell lines (from swine, cattle and sheep) carry introduced TERT or SV40 genes [69], which results in regulatory challenges. Therefore, the third strategy of selecting fast‐growing cells seems to be a promising approach at the moment. In contrast to differentiated cells, stem cells have high proliferation rates and can be cultivated for extended periods of time. Several types of stem cells are commonly used in research: embryonic stem cells (ESCs), induced pluripotent stem cells (iPSCs) and mesenchymal stem cells (MSCs) or satellite muscle cells (SMCs). ESCs are obtained from the inner cell mass of blastocysts and can be differentiated into any cell type, but their use is controversial and not widely accepted by investors and end users. iPSCs also have high differentiation capacity, but are created through genetic engineering techniques, which raises concerns among customers regarding the principles of sustainable food production—they are viewed as a genetically modified organism with subsequent regulatory and marketing challenges. One more approach to reprogram somatic cells is the treatment with a cocktail of small molecules—it was demonstrated that pluripotency can be chemically induced in cells of different origin in this way (Chemically induced PSCs—CiPSCs) [70, 71]. MSCs isolated from adult tissues can be easily expanded in vitro and differentiated into several cell types, including bone and fat. SMCs, myogenic progenitor cells derived from skeletal muscle tissue, can be easily differentiated into muscle cells.

### 3.2 Media composition

Almost all mammalian cell expansion protocols use cell culture media, which consists of basal media to provide cells with glucose, amino acids, physiological pH and osmolarity, and serum supplements which provide biological signal molecules, growth factors, hormones, and adhesion factors. Because standard **serum supplements** are of animal origin, and the most used is FCS, derived from the blood of unborn fetuses, their use raises ethical animal welfare concerns. Alternatively, **recombinant proteins** can also be used as alternative to standard serum. In fact, several serum‐free media are already available on the market, but such media require an additional coating of the cell growth surface (mostly xenogeneic proteins).

Since serum supplements and recombinant proteins are exhausted in media during a cultivation, new fresh media is regularly exchanged. Medical grade culture media costs are around 400$/L, which makes the whole process very expensive. In order to achieve an affordable cultivated meat production, both the medium itself and the medium exchange protocols must be optimized. There are several strategies to reduce the cost of production. For example, (1) recycling the spent medium after removing toxic metabolites (lactate, ammonia)—it has been shown that 50% of the amino acids are still present in the cell culture medium waste; (2) analyzing the media composition in real‐time to monitor the use of media components by the cells and quickly replace depleted components without a full media exchange; (3) using food‐grade instead of pharma‐grade media components; (4) replacing amino acids produced individually by fermentation by plant‐based protein hydrolysates [72] or (5) finding new bioactive plant molecules to support cell growth and differentiation or use natural bioactive compounds with antimicrobial effects instead of antibiotics and antimycotics [73].

### 3.3 Expansion strategies

Most types of mammalian cells belong to an anchorage‐dependent cell type, requiring a growth surface or rely on cell‐to‐cell interactions to proliferate ex vivo. The anchorage dependency causes surface area limitations since it is not easily scalable. In general, there are three ways to bring anchorage‐dependent cells to suspension cultivation: (1) cultivation on microcarriers, (2) as cell aggregates and (3) cells encapsulated in hydrogels. To avoid using proteases for harvesting (used to recover cells from the surface of microcarriers), cells can be cultivated on edible microbeads (e.g., soy, zein). In this case, the choice of the material of beads must be carefully considered since they can affect the sensory properties of the final meat product such as taste, color and texture. 2.9 × 10^11^‐8 × 10^12^ cells are required to recover 1 kg of protein from muscle cells, which would require a stirred tank bioreactor (STR) in the order of up to 5000 liters [74, 75]. Stirred tanks are one of the most widely used types of reactors due to their relatively simple structure, scalability and well‐characterized properties. However, Computational Fluid Dynamics (CFD) simulations of STR cultivation with microcarriers revealed the presence of oxygen and shear stress gradients in 203 m bioreactor, while even more hydrodynamic stress is introduced via sparging [76]. Air‐lift bioreactors, another type of **mechanically driven** bioreactors are also discussed as candidate platform for mammalian cell agriculture [77]. Further mechanically driven reactor types suggested suitable for mammalian cell expansion are vertical wheel bioreactors and wave‐reactors. Other options are **hydraulically driven bioreactors** (e.g., hollow fiber bioreactors and fixed‐bed bioreactors) containing fixed fibers or scaffolds for cell adhesion while media is pumped to flow along those structures seeded with cells. Hollow fiber bioreactors consist of numerous semi‐permeable hollow fibers placed in a cylindrical reservoir. Cells can be seeded inside the fibers while medium constantly flows along their external surface. Alternatively, the opposite seeding principle is available for some models: cells in medium are seeded on the external surface of the hollow fibers while oxygenated medium flows inside them. Moreover, several units can be connected in parallel which enables a scale‐up principle. On the one hand, cultivation in hollow fiber reactors does not require the use of microcarriers, on the other hand, the longitudinal concentration gradient forms when the medium flows along the fibers and can thus lead to an uneven distribution of nutrients. Cell harvesting can also be problematic in such systems, as dissociation reagents cannot efficiently penetrate the 3D‐growing cell mass.

Feeding strategies are also a complex issue, as an acceptable balance must be achieved to reach cost‐effective production without compromising cell proliferation and viability. In routine 2D cell cultures in the lab medium is completely exchanged every 3 to 4 days in order to keep high cell viability. However, amino acids and glucose are not completely consumed in this time period. Thus, partial media removal can be applied to reduce operating costs [78]. Accumulation of toxic metabolites in growing mammalian cell cultures is a serious limitation, since concentrations of 2–10 mM NH_3_, for example, are inhibitory [79]. The accumulation of lactate affects proliferation indirectly by lowering pH and is directly toxic to cells at certain concentrations. In the batch and fed‐batch process, toxic concentrations are reached faster than in perfusion systems, but perfusion systems require higher volumes of media.

The maximum cell density supported in an STR is limited by culture viscosity, gas‐liquid mass transfer rates, catabolite accumulation rates, mixing time, and other factors [72]. Therefore, it is economically more efficient to stop the batch when the limits are exceeded, and the growth rate is reduced and start a new batch with unlimited growth. In simulations for 20 m^3^ bioreactors, cell mass production in the fed‐batch process leads to a **total cost** of 37 USD/kg wet cell mass and for the perfusion process to 51 USD/kg wet cell mass [79]. Cost optimization via, for example, substitution of amino acids with plant protein hydrolysates in a fed‐batch process could potentially bring costs below 25 USD/kg [79]. At least as affordable “sometimes” food, cultivated meat should reach the target price of no more than 50 USD/kg (the price of a premium cut of meat). If the production of wet cell mass costs USD 25/kg, with further processing, packaging and distribution another USD 25/kg, the displacement of conventional meat by cultivated meat in terms of the price is currently still not advantageous, since the organoleptic and nutritional properties of the cultivated cells are at the time not yet comparable to those of normal meat [79]. Possible strategy here would be hybrid (plant protein plus cultivated cells) products.

### 3.4 Scaffold materials

Scaffolding materials for cultivated meat production are also being intensively investigated. Edible materials can be used here both as **microcarriers** during expansion and as **scaffolds for textured meat** to create attractive meat analogues. The application of scaffold materials depends not only on their composition, but also on the final properties such as porosity, biodegradability, tunability and stiffness. The material porosity will allow cell penetration into the scaffold, as well as nutrition supply during cultivation process [80]. Tunability and stiffness will directly influence not only cell growth and differentiation, but also final product characteristics. Various materials can be used as microcarriers or scaffolds: polysaccharides—alginate, starch, chitosan, pectin (although they do not contain special motifs for cell adhesion); polypeptides and proteins—collagen, elastin, gelatin (but not from animals!), gluten; lipids—paraffin, shellac (may bring oily flavors); synthetic—PGA, PEG. Complex natural components such as mycelia and decellularized plant tissue are also very attractive candidates.

Edible patterned films (for muscle cell alignment into myotubes) fabricated from gelatin, alginate, konjac, soy, glutenin, zein and chitosan were also reported [81]. The main issue here is not the materials themselves (however, they must not affect the taste of the final meat product), but the size/thickness of the scaffolds, which can affect cell viability in the absence of oxygen and nutrient delivery systems (vascularization). Average steak thickness (from New York strips to fillet mignon) is 3.8 cm and diffusion of, for example, oxygen into tissues is believed to be limited to up to 200 μm [82]. This means that creation of the bulk meat structures requires either scaffold vascularization (which presents a challenge) or active perfusion (which can compromise cell viability through shear stress). Alternatively, scaffolds with perfusion channels can be fabricated.

### 3.5 Differentiation

To generate muscle or fat cells, stem or progenitor cells must undergo differentiation and maturation. Standard differentiation protocols, developed for regenerative medicine include the use of differentiation factors of different nature, including hormones and small (sometimes toxic) molecules. For instance, adipogenic differentiation is usually triggered by the addition of medical‐grade compound ds such as **dexamethasone** (a glucocorticoid with anti‐inflammatory and suppressing effect on the immune system) which is thought to activate the C/EBP family of adipogenic transcription factors. Another component of adipogenic cocktail is 3‐isobutyl‐1‐methylxanthine (IBMX), nonselective phosphodiesterase inhibitor, that results in an increase in cellular cyclic AMP levels and activation of protein kinase A. Next component, **insulin**—a lipogenic hormone which activates metabolic processes of glucose and lipogenic responses, enhancing fatty acid and triglyceride synthesis in adipocytes and other cells [83]. **Thiazolidinediones** are a drug class of specific PPARγ agonists which is often used to significantly enhance adipogenesis in many cell types. Although the listed substances are approved for treatment of different diseases and conditions, they are not approved for human consumption. A similar scenario can be observed in the context of myogenic differentiation, wherein the utilization of hydrocortisone and dexamethasone is employed within the differentiation media. To address this issue, researchers are exploring the potential of natural plant substances to induce differentiation. For instance, Chinese medicinal herbs such as Dan phenolic acid B, icariin and astragalosides are being tested. Additionally, specific protocols involving fatty acids are being developed for adipogenic differentiation [84].

The chemical and physical properties of scaffold materials also play an important role in stem cell differentiation. Most stem cells are mechanosensitive, meaning that these cells can recognize mechanical environmental signals and convert them into intracellular biological signals. The ability to selectively design the properties of scaffold materials is a smart approach that can help complement or potentially even eliminate the use of exogenous differentiation factors. It could be shown that the chondrogenic and osteogenic differentiation can be influenced by the material composition [85]. Chemical factors such as glucose and oxygen concentration are also important regulators of cell differentiation [80].

Another problem is **simultaneous differentiation for the different types of cells** which are seeded on the scaffold. It is difficult to develop differentiation factor cocktails, which for example trigger the adipogenic differentiation in one cell type and do not affect myogenic differentiation in another cell type. Potentially, the start of the differentiation into different directions can be separated in time or spatially controlled by immobilizing the differentiation factors in the scaffolding material.

### 3.6 Regulatory challenges and acceptance

The first important point of the regulation is the terminology—farmers in US and Europe are betting on a new law that will allow only products derived from animals to be called “meat”. And if cultivated meat is not meat, should it be regulated the same as conventional meat? [86] A European Union regulatory framework for cultivated meat was established already in 1997 and updated in 2018 [7]. If cells or organisms have not been genetically modified, the product will be governed by the EU Novel Food Regulation. Genetically modified cells (e.g., iPSCs) are subject to EU GMO legislation. General point of regulations is that the product is safe, wholesome and unadulterated [7]. For authorization under the EU Novel Food Regulation, information should be provided on the identity of the product, the manufacturing process, the composition, the proposed use, the amount used and the expected intake of the product. Additional information should include allergenicity, nutritional and toxicological properties, as well as absorption, distribution, metabolism, and excretion. The European Food Standards Authority gives its opinion to the European Commission and compares this food with a similar category that exists on the EU market.

The next important point is consumer behavior towards cultivated meat, as consumer acceptance is critical for the market success of cultivated meat. Since, to date, only Singapore (and newly US) offers a market for cultivated meat, it is very difficult to analyze and assess the demand and consumer behavior regarding in vitro cultivated meat products. However, Bryant and Barnett (2018 and 2020) conducted meta‐analyses on consumer acceptance, which showed that the ratio of possible consumers varies significantly between 5 to 11% to 65% depending on country and group of respondents [87, 88, 89]. Showing that on average especially young, more educated and liberal consumers would be interested in cultivated meat. Other studies show, that urban groups accept cultivated meat more quickly than rural ones, and men are more likely to try it than women [90, 91]. A comprehensive review of consumer behavior revealed that the majority of consumers would currently be willing to try cultivated meat, but would not necessarily substitute it for traditional meat on a daily basis [92]. Surprisingly, meat industry workers who are directly involved in meat production show a high acceptance of cultivated meat. Geographical analysis does not allow for clear correlations with food cultures, but for example customers in the US show higher acceptance (40%–60%) compared to some Asian countries (25%–35%, excluding Vietnamese at 54%) or the UK (18%). In China, 60% of survey participants are very likely to buy cultivated meat [93].

Siddiqui et al. described fears, conspiracy theories, conservatism, phobias, disgust, scepticism and scientific mistrust as main arguments and factors against eating cultivated meat [92]. Additional obstacles are price and sensory expectations [89]. The perceived unnaturalness of cultivated meat is the biggest psychological barrier to its use, shown in several studies where customers experienced significant anxiety [92]. Customers expect worse texture, flavor and look of cultivated meat and anticipated it as “boring”. A recent study discovered that customers are less disgusted eating insects than trying cultivated meat [94]. In addition to taste, price is another product characteristic that will be decisive for the market success of cultured meat. To increase acceptance, product communication and marketing should strive to present cultivated meat in a more natural and beneficial light (as confirmed by the public tasting of the first cultivated hamburger described above).

## 4 CONCLUDING REMARKS AND PERSPECTIVES

The cultivated meat market is experiencing rapid growth and attracting significant investments, including from governments. However, several challenges still need to be addressed to fully realize its potential. These challenges include media optimization, sensory properties, scalability, and price. Unlike the pharmaceutical industry, it is essential to reduce production costs by achieving a cost‐effective scale‐up of production processes to make cultivated meat commercially viable. Currently, it is not feasible to completely replace traditional livestock farming or meet the growing demand for meat worldwide only with cultivated meat. Promising strategies, such as developing hybrid meat analogues that combine plant proteins with animal cells, or developing food‐grade expansion and differentiation media with molecular farming are constantly showing solutions to the mentioned bottlenecks. Moreover, the acceptance of cultivated meat from two startups in the USA in May 2023 determines a breakthrough bringing the first consumer feedback from a 400 M people market. Overall, a significant proportion of consumers are open to trying cultivated meat, but there is a need for increased communication and outreach to enhance acceptance. Continued efforts in educating the public and addressing any concerns or misconceptions surrounding cultivated meat will play a vital role in its widespread adoption.

## CONFLICT OF INTEREST STATEMENT

Marline Kirsch and Jordi Morales‐Dalmau are employees of Cultimate Foods UG (Berlin/Göttingen). However, the authors declare that they wrote this review objectively and without bias. The company did not influence manuscript preparation. No other conflicts of interest exist.

## Data Availability

This manuscript does not report data.
